# The long and the short of Periscope Proteins

**DOI:** 10.1042/BST20220194

**Published:** 2022-10-05

**Authors:** Fiona Whelan

**Affiliations:** Department of Molecular and Biomedical Science, The University of Adelaide, South Australia 5005, Australia

**Keywords:** adhesin, bacterial surface proteins, genotypic variation, invasin, tandem repeat

## Abstract

Bacteria sense, interact with, and modify their environmental niche by deploying a molecular ensemble at the cell surface. The changeability of this exposed interface, combined with extreme changes in the functional repertoire associated with lifestyle switches from planktonic to adherent and biofilm states necessitate dynamic variability. Dynamic surface changes include chemical modifications to the cell wall; export of diverse extracellular biofilm components; and modulation of expression of cell surface proteins for adhesion, co-aggregation and virulence. Local enrichment for highly repetitive proteins with high tandem repeat identity has been an enigmatic phenomenon observed in diverse bacterial species. Preliminary observations over decades of research suggested these repeat regions were hypervariable, as highly related strains appeared to express homologues with diverse molecular mass. Long-read sequencing data have been interrogated to reveal variation in repeat number; in combination with structural, biophysical and molecular dynamics approaches, the Periscope Protein class has been defined for cell surface attached proteins that dynamically expand and contract tandem repeat tracts at the population level. Here, I review the diverse high-stability protein folds and coherent interdomain linkages culminating in the formation of highly anisotropic linear repeat arrays, so-called rod-like protein ‘stalks’, supporting roles in bacterial adhesion, biofilm formation, cell surface spatial competition, and immune system modulation. An understanding of the functional impacts of dynamic changes in repeat arrays and broader characterisation of the unusual protein folds underpinning this variability will help with the design of immunisation strategies, and contribute to synthetic biology approaches including protein engineering and microbial consortia construction.

## Introduction

### Fibrillar proteins at the dynamic bacterial cell surface

The bacterial cell surface is a variable landscape, featuring dynamic changes in the export of biofilm components [1,2]; surface modifications to the cell wall [3] and surface proteins [4]. Variability confers adaptability to changes in the bacterial environment, for example, to attach to host cell surfaces [5]; establish a bacterial biofilm community [6,7]; regulate bacterial motility [8]; and conceal antigens from the immune system of the host [9]. Surface proteins important for regulating niche adhesion and forming cell–cell contacts are necessarily vulnerable to environmental insults due to their exposure, thus bacteria have evolved robust proteinaceous filaments supporting these bacterial surface activities, including pili, fimbriae and curli. Gram-negative bacteria build micrometre scale pilus assemblies that can be dynamically assembled by complex membrane-associated chaperone machinery (reviewed in [10]); the second group of fibrillar surface proteins, typically Gram-positive examples, extend on nanometre length scales and are assembled as homopolymers of globular domains decorated with functional tip domains, e.g. adhesin domains, where export machinery covalently link globular domains together, featuring spontaneous isopeptide bonds to stabilise extended topologies (reviewed in [11]). The third group of small fibrillar proteins require no assembly, comprising a single polypeptide chain attached at one end to the cell wall, and forming rod-like structures ‘stalks’ that can project functional domains tens of nanometres from the cell. Observations of variability in the size of these fibrillar proteins between closely related bacterial strains have been described over several decades [12–16]. These apparently uncomplicated single-chain rods conceal an elegant adaptive mechanism underpinned by the presence of a series of highly repetitive sequence motifs in the stalk region, a defining feature of ‘Periscope Proteins’. The Periscope Protein class includes monomeric single-chain proteins with highly identical tandem repeat domains that can fold individually, and in the context of arrays, form anisotropic rod-like structures. Notably, this definition excludes Gram-negative multimeric stalk proteins such as the trimeric autotransporter adhesin (TAA) family, which lack tandem repeat domains, instead incorporating modular polymeric alpha-helical parallel coiled coils interspersed with connector domains, which have been observed to vary in repeat sequence identity and feature highly mosaic structures [17–19]. However, a similar function to the Periscope Protein class may be mediated by the TAA family, where variation in peptide repeat number has been correlated with changes in surface exposure of the cell-distal head domains, impacting biofilm formation, adhesion and immune evasion [20,21].

### Rod-like monomeric fibrillar repeat proteins

**Figure 1.**
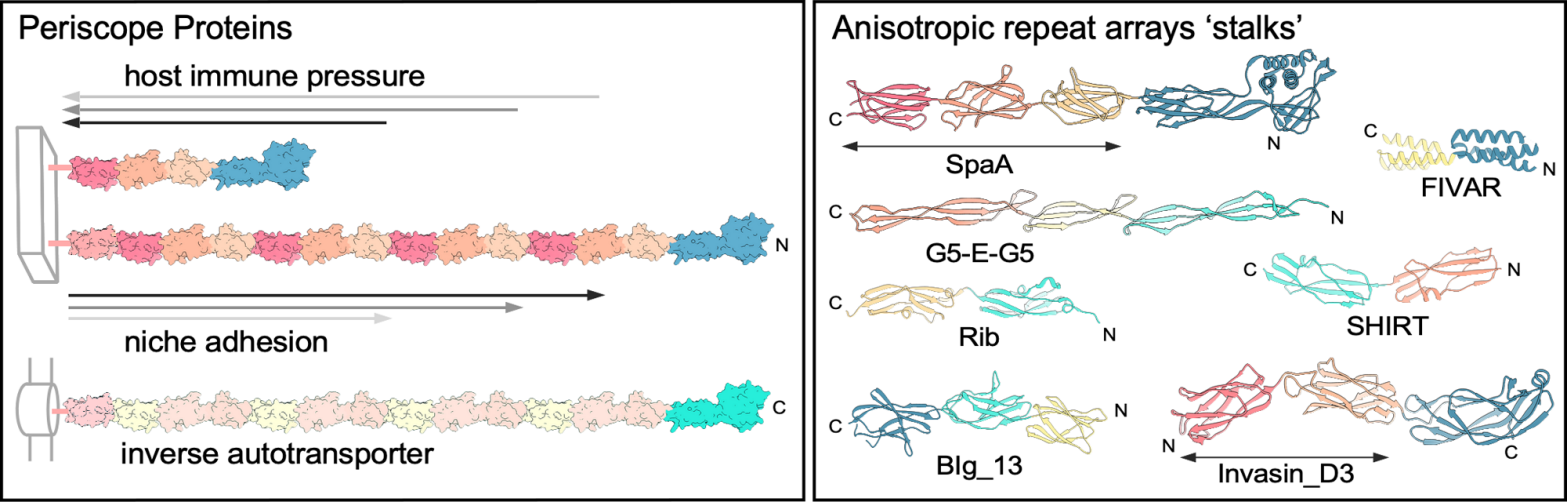
Periscope Proteins and examples of tandem repeat ‘stalks’. Periscope Proteins in Gram-positive organisms are typically cell wall attached via sortase cross-linking at the C-terminus and feature a repetitive ‘stalk’ (pink, red, orange) that projects an N-terminal adhesin domain (blue) out from the cell surface. Gram-negative Periscope Proteins include inverse autotransporters, with an outer membrane-embedded β-barrel translocation unit, a repetitive stalk (pink, yellow) and C-terminal extracellular effector (green) (illustration adapted from PDB accession: 6fwv [75]). Host immune pressure has been demonstrated to select for shorter repeat tracts in Periscope Proteins, while niche adaptation via adhesive interactions have been proposed to select for longer repeat tracts with greater projection of effector domains. Tandem repeat folds (right) illustrate anisotropic ‘stalk’ arrays typical of this protein class, including Pfam families: SpaA (Pfam family PF09134, PDB accession: 6fwv [75]); FIVAR (PF07554, 4kjm, unpublished); G5-E-G5 (PF17041/PF07501, 4wve [26]); Rib (PF08428, 6s5y [24]); SHIRT (PF18655, 7avh [25]); BIg_13 (PF19077, 2yn3 [76]); and Invasin_D3 (PF09134, 5ldy [77]). Protein structures were rendered in Chimera.

A series of studies focused on determining the fold and function of highly repetitive protein sequence motifs of the Periscope Protein class led to the identification of a propensity for the formation of rod-like structures. Following the empirical definition of folding boundaries, X-ray crystal structures of individual repeat domains and tandem repeat units suggested these multi-domain constructs were anisotropic, having large differences in their axial dimensions (e.g. adhesins SasG [22]; Aap [23]; Rib [24]; and Sgo0707 [25]). Light scattering experiments [24–26]) and high-resolution fluorescence single-molecule imaging to measure intramolecular distances [25,26] defined the formation of long highly anisotropic folds that were indeed rod-like in solution, with a step-size correlated with repeat number [26]. Simulation of the folding behaviour of large numbers of tandem SHIRT repeats supports the formation of highly extended rods [25]. Extreme anisotropy is maintained by high-stability domain folds that exploit different mechanisms to maintain an elongated trajectory, including (i) very short rigid linkers between globular domains (Rib, Sgo0707); or (ii) intimate interdomain contacts (SasG, Aap) supporting formation of rods on scales of ∼2 nm wide and tens of nanometres in length [26,27] (Figure 1). Unusually high melting temperatures (*T*_m_) (Sgo0707 *T*_m_ 75.9°C; Rib *T*_m_ 71°C) and fold tensile strengths (SasG E domain 250 pN; G5 domain 420 pN, at 800 nm s^−1^ retraction rate [26]; Aap E domain 312 pN and G5 domain 475 pN, at 1000 nm s^−1^ [28]) are predicted to support the maintenance of the folded state and extension of adhesin domains from the cell surface, establishing a robust adhesive tether.

Preliminary observations suggested that a key feature of these unusual fibrillar repeat proteins is the maintenance of high identity at the level of the coding sequence between tandem repeats. Observation of limited sequence identity between neighbouring tandem repeats in mammalian systems of 30–40% [29]; and a tendency for tandem repeats with very high sequence identity with form stable misfolded structures [30], highlights the novelty of these bacterial surface rod-like repeats. What is the selective advantage gained for these bacteria, given the extant risk of misfolding; and how widespread is this phenomenon? Here I review the discovery, structural characterisation and diverse functions of the bacterial Periscope Protein class.

## Discovery of the Periscope Protein class

Fluctuating numbers of tandem repeats in bacterial cell surface proteins have been observed in related bacterial strains and protein homologues over decades of research, hinting at a widespread mechanism of bacterial surface variability. Host immune pressure has been observed to generate variable numbers of repeats in Group B *Streptococcus* (GBS) adhesin Rib, impacting pathogenicity and immune evasion [31] (Figure 1). Repeat number variation in *Staphylococcus aureus* adhesin SasG (3–13 repeats) [25,32], and homologous *Staphylococcus epidermidis* adhesin Aap (3–17 repeats) [33] has been observed, where SasG variability is correlated with modulation of ligand binding by other surface-associated proteins [34], a process implicated in bacterial dissemination in the host. Variation in repeat numbers has also been identified in a range of other species, including *Enterococcus faecalis* biofilm-forming protein Esp [35]; *Pseudomonas aeruginosa* extracellular exopolysaccharide binding protein CdrA [36]; and many Gram-negative inverse autotransporters (Figure 1) (reviewed in [37]) of the intimin/invasin family of adhesins, including extremely variable numbers of bacterial immunoglobulin-like (BIg) repeats (2–47 repeats) [38].

Characterisation of the unusual structural topology and domain stability of fibrillar tandem repeat proteins spurred bioinformatics approaches for the discovery of novel stable domains selected by this harsh functional niche. A search for coding sequences with similar sequence motif patterns was undertaken, looking for genes encoding tandem repeats of >50 residues with high sequence identity between repeats. Short-read sequencing approaches are unable to accurately resolve repeat elements [39], thus the long-read ‘PacBio’ sequence database National Collection of Type Cultures 3000 was interrogated to identify these coding sequences in highly related pathogenic bacterial strains. This work identified >1500 proteins containing highly identical repeats, defining a diverse series of characterised folds and new Pfam families [40] predicted to form rod-like topologies associated with fibrillar proteins from both Gram-positive and Gram-negative bacteria. These proteins were clustered into 180 groups, with 84 having repeat number variations ranging from small differences (e.g. 4–6 PATR repeats (Figure 1), β-helical fold, *Salmonella enterica* autotransporter adhesin ShdA) to extreme examples of repeat number variability (2–46 repeats, *Streptococcus agalactiae* SraP), correlated with DNA repeat identity [25]. A subset of these variable repeat number proteins do not feature cell-wall attachment motifs, incorporating domains associated with enzymatic functions in peptide and oligosaccharide degradation and cell wall remodelling (e.g. *E. faecalis* Autolysin (Uniprot: A0A2Z6BTL7); *Clostridium perfringens* Lysozyme LytD (Uniprot: A0A2X2YDI6); *Streptococcus gordonii* Autolysin (Uniprot: A0A2L2PB53); the function of repeat variability for these enzymes is not yet defined [25].

### Tandem repeat domain folds and interdomain linkers

Focusing on cell-wall attached proteins, folds identified in the tandem repeat regions of the Periscope Protein class include domains of ∼50–165 residues in length, including β-sandwich folds of the immunoglobulin-like superfamily E-set (Pfam class: CL0159); β-grasp ubiquitin-like folds (CL0072); three-helix bundles (CL0589); and some new domains identified within those groups including all-β Ig-like CshA_repeat (Pfam accession: PF19076); bacterial Ig-like BIg_13 (PF19077); and a repeat domain of unknown function DUF5801 (PF19116), among others (Figure 1). A subset of structurally characterised domain folds has been interrogated [25] to determine the angular offset and distance of the N- and C-termini, where angles of ∼180° and longer inter-terminal distances would be expected for linear repeat arrays. From this analysis, the length of interdomain linkers correlated with the angle and distance of the N- and C-termini; for those tandem repeats with a near 180° angular offset of termini, separated by a larger distance, the linkers typically comprise 5 residues or less and often feature Proline residues, associated with increased rigidity. The corollary was also observed, where longer and more dynamic linkers were observed between tandem repeats with smaller angular offset and decreased distance between termini, e.g. ∼34° angle and only 5 Å distance for the *E. faecalis* Autolysin LysM repeats (Uniprot: P37710), potentially conferring increased flexibility for tertiary array formation [25]. A potential role for flexible repeat arrays has been proposed for *S. gordonii* CshA, with the observation of a combination of folded and partially folded repeats, possibly supplying flexibility for optimal projection of the adhesive tip domain [27]. The *Escherichia coli* virulence-associated inverse autotransporter intimin deploys flexible and rigid linkers between BIg domains. This combination has been proposed to play a complementary role to increase the radius of reach of the cell-distal lectin-like adhesin domain, and to optimise the orientation for interaction with the mammalian host cell [41].

## Periscope Proteins — length variability and immunogenicity

**Figure 2.**
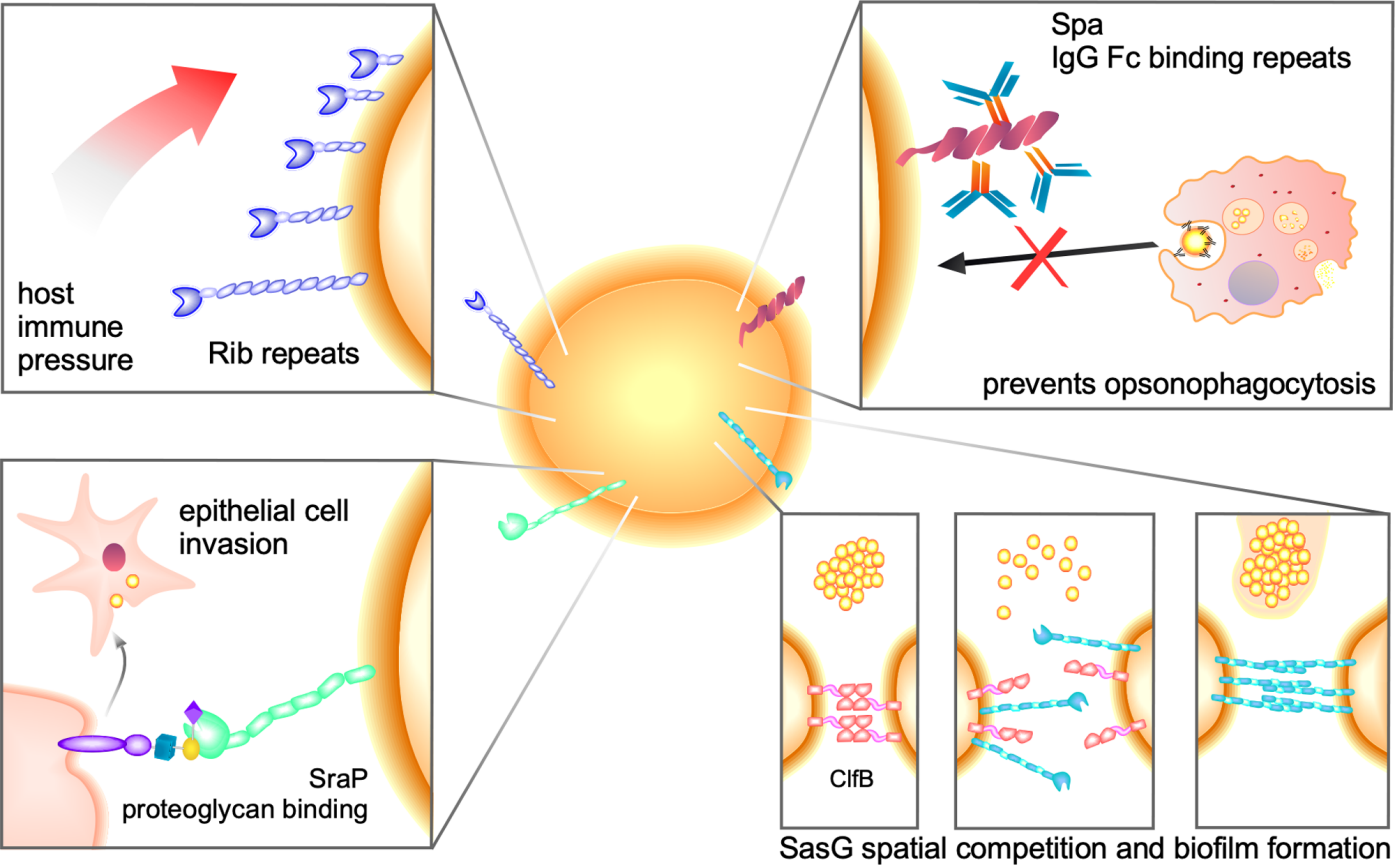
Diverse functionality of the Periscope Protein class. Periscope Proteins project from the bacterial cell surface; for example a Gram-positive coccus. Selection of decreased tandem repeat tract size has been demonstrated for Rib repeat adhesins following exposure to host the host immune system (top left). *S. aureus* Spa binds to the constant Fc region (orange) of IgG antibodies (blue/orange) to prevent IgG hexamer formation, opsonisation and phagocytic killing (top right). Periscope Protein invasins bind to terminal glycan branches e.g. Neu5Ac (*N*-acetylneuraminic acid, purple diamond) of proteoglycan receptors (purple) to initiate cellular uptake of bacterial cells, e.g. SraP (green) facilitates the uptake of *S. aureus* by epithelial cells (bottom left). SasG (light blue) interferes with Clumping factor B (ClfB, pink) dependent *S. aureus* clumping, with a minimum requirement for 5 E-G5 repeat units (bottom right). Proteolytic removal of the adhesin domain of SasG supports the formation of higher-order SasG assemblies and protein-dependent biofilm formation that is similarly length-dependent (bottom right).

The observation of variability in repeat number in the context of rod-like protein topology is thus predicted to correlate with variation in the distance the N-terminal functional domain is projected from the cell by the repetitive stalk, typically terminating in a C-terminal cell-wall cross-linking motif (Figure 1). Inspired by the rod-like topology of these surface proteins, the memorable ‘Periscope Proteins’ class was coined to capture the concept of population level extension and retraction of functional rod-like proteins from the bacterial cell surface. Given the very high sequence identity between tandem repeat coding sequences, it has been proposed that the likely mechanism of variation is homologous recombination (reviewed in [42]). Analysis of the location, function and gene ontology (GO) terms assigned to Periscope Proteins reveal key roles for this class of protein that can broadly be categorised as a combination of immune modifying, invasive or adhesive (Figure 2). Functional domain folds are typically located at the N-terminus, with cell wall tethers at the opposite end of the molecule hypothesised to anchor the protein rods to the cell wall, likely conferring variable exposure of the functional domain at the surface of the bacterium. This class includes adhesins, invasins, inverse autotransporters, and biofilm-forming proteins, fulfilling a range of surface functions in adhesive interactions and bacterial virulence. Notably, the mechanistic implications of variable repeat numbers remain to be determined in many cases; additionally, limited research focused on the repeat regions themselves has defined functional roles beyond mere anisotropic scaffolding, highlighting the need for more direct interrogation of the function of repetitive ‘stalks’.

A large body of literature describing the impact of Periscope Protein repeat variability on the Streptococcal family of Alpha-like proteins (Alp; Alpha C protein, Alp1—Alp4, R28 and Rib proteins) has correlated virulence and antigenicity with Rib domain repeat number (reviewed in [43]). Highly polymorphic tandem repeat numbers in surface protein adhesin R28 have been identified in a large sample of isolates of invasive *S. pyogenes* infections in the human host, with 1–17 Rib repeats evident [44]. The expression of R28 is correlated with human epithelial cell adhesion [45] and virulence [44]. Mouse and non-human primate models of M28 *S. pyogenes* necrotising myositis have demonstrated increased lesion size and decreased survival rates for animals infected with strains of M28 that express higher levels of *R28* transcript and R28 protein [44]. Hypervirulent clonal complex CC17 GBS, overrepresented in GBS-infected newborns, adapts to host immune pressures over time by changing components of the bacterial cell envelope, including a marked reduction in the size of the Rib domain repeat region of Rib protein at ∼1 month postpartum [46]. Mouse models of infection following passive immunisation with antisera against *S. agalactiae* surface repeat protein Alpha C demonstrate reduced tandem Rib domain repeat number from 9 down to 1–4 repeats (Figure 2), correlated with reduced susceptibility to antibody-mediated opsonophagocytic killing [47]. Thus, truncation of repeat numbers through homologous recombination may effectively withdraw the antigen from immune surveillance; or generate antigenic variation [31].

A seemingly antithetical role for repeat number variation has also been observed for *S. agalactiae* Alpha C, where increased repeat numbers were found to decrease the overall immunogenicity of the full-length protein, indicating a potential role in the evasion of host immunity [48]. A complex role for immunodominance of the repeat region has been investigated in *S. aureus* Staphylococcal protein A (Spa), where Ig-binding domain repeats (Pfam accession: PF02216) bind the Fc region of IgG antibodies, preventing complement deposition and opsonophagocytosis (Figure 2) [49]. Spa also acts as a superantigen with a bias towards binding variable heavy 3 idiotype B cells ∼50% of mature B cells in human adults ([50] and references therein), inducing B cell programmed cell death [51], limiting host immune responses to other subdominant antigens that are necessary to prevent recurrent infection [52]. With the observation of the immunodominance of the repetitive regions of Rib and Alpha C [53], protein-based vaccine strategies excluding the repetitive ‘stalk’ have generated a Rib/Alpha C N-terminal domain fusion protein (GBS-NN) now in clinical trials for the prevention of invasive neonatal disease [54].

## Invasins, adhesins and biofilm factors

Selection pressures generating increased repeat numbers have not been identified, however, positive selection for exposure of niche-specific invasins, adhesins, or biofilm factors has been proposed to explain this phenomenon [14], although the effect of variation in repeat numbers on function remains elusive. Adhesive and biofilm-forming functions are attributed to a range of protein folds, including, for example, bacterial lectin-like domains associated with host glycan and proteoglycan binding [55,56]; collagen-binding domains for attachment to the host connective tissues [57]; and fibronectin-binding domains for extracellular matrix adhesion [58,59].

A broad array of such functional domains implicated in host cell invasion, adhesion and biofilm formation have been identified within the Periscope Protein class; in some cases, the repeat domains within the stalk region directly affect these functions. Epithelial cell internalisation of *S. agalactiae* is mediated by an interaction of the N-terminal adhesin domains of Alpha C protein with α1–β1-integrin [60], and/or glycosaminoglycan [61]. The N-terminal legume-like lectin domain of SraP binds to the terminal proteoglycan moiety N-acetylneuraminic acid, supporting *S. aureus* invasion of host epithelial cells [55] (Figure 2). *Pseudomonas aeruginosa* biofilm matrix protein CdrA binds to exopolysaccharides to stabilise the aggregate structure under fluid shear, with the most profound impacts of knockouts evident for those strains with the largest numbers of tandem repeats [36]. Cryo-electron tomography imaging of *P. aeruginosa* PAO1 CdrA (Uniprot accession: Q9HVG6) at the cell surface determined a mean protein length of 71 nm (15 MBG_2 repeats), with the formation of CdrA:CdrA cell–cell junctions stabilised by polysaccharide binding to establish antibiotic-resistant biofilm [62]. The non-repetitive cell-wall distal N-terminal region of *E. faecalis* biofilm-associated surface protein Esp forms pH-dependent amyloid-like fibrils, demonstrated to incur cell clumping behaviour in heterologous expression strains [63]. Staphylococcal von Willebrand factor (vWF)-binding protein Vwbp binds to vWF under shear stress, contributing to blood vessel adhesion [64], initiation of endocarditis, and is a virulence determinant in joint-invasion and septic arthritis ([65] and references therein), although the molecular mechanism of binding has yet to be determined. Platelet binding mediated by *N*-acetylneuraminic acid binding Siglec-like domains has been identified in *Streptococcus oralis* subsp. *oralis* Periscope Protein AsaA featuring 28–31 DUF1532 repeats and a C-terminal cell-wall attachment motif. AsaA contributes to infective endocarditis vegetation colonisation in a rabbit model of infection, with homologues identified in *Gemella haemolysans*, *Granulicatella elegans*, *Staphylococcus pasteuri* and *Streptococcus mitis* featuring varying numbers of DUF1532 repeats [66].

Direct roles for the tandem repeat or ‘stalk’ regions in Periscope Proteins have been identified for many different domain folds. The E-G5 tandem repeat regions of SasG and Aap mediate protein-dependent biofilm formation following truncation or cleavage of distal adhesin domains [67,68] (Figure 2); while isolation of oligomeric assemblies of the repeat region of Aap with titration of ZnCl_2_ [69] infers a potentially broader role for repetitive rod-like proteins in homotypic cell–cell tethering. SasG-dependent biofilm formation was also demonstrated to be length dependent, where five E-G5 repeats (≥50 nm in length) [26] were sufficient to establish protein-dependent biofilm [34] (Figure 2). Streptococcal surface repeat (SSURE; Pfam accession: PF11966) domains of *Streptococcus pneumoniae* PavB bind directly to human Thrombospondin-1, supporting adhesion to host cells and the extracellular matrix [70]. Periscope Proteins functioning as anti-adhesive factors have also been observed in the case of *S. aureus* proteins Pls [71] and SasG [34]. In a rare example of an explicit study of the effects of repeat number variation on function, surface-associated *S. aureus* host binding proteins clumping factor ClfB and fibronectin-binding protein A (FnBPA) demonstrate length-dependent interference by SasG [34], where repeat regions less than 4 E-G5 repeats (∼40 nm) have no effect, but 5 E-G5 repeats or more (≥50 nm in length) [26] are able to block binding to host ligands, inferring variable repeat numbers incur a functional outcome related to spatial competition at the cell surface (Figure 2). A potential function for variation in repeat numbers has been identified in the functionally related TAA family. The length of a surface adhesin, YadA, has been demonstrated to be correlated with the length of the needlelike Type III secretion ‘injectisome’ from *Yersinia enterolitica*, where spatial coevolution is required for injectisome secretion of effector proteins into host cells [72]. Whilst this example is not formally a Periscope Protein, it is possible that similar spatial covariance strategies are explored within the panoply of surface adhesins of the Periscope Protein class.

## Future direction

From these observations, it appears Periscope Proteins have dynamic functional roles at the population level, mediated by both the distal adhesin domains and directly by tandem repeats, including regulation of cell–cell contacts; host cell invasion; niche adhesion; and limiting immune clearance. Additionally, bioinformatic approaches to the discovery of new bacterial surface repeat proteins based on known rod-like repeat and adhesin folds [73]; and deploying a machine learning discovery approach [74], have identified >6000 new bacterial surface repeat proteins implicated in adhesion and possibly catalysis. However, tandem repeat domain variability is yet to be characterised in this dataset. As more strain and substrain genomic data becomes available through long-read sequencing projects, we anticipate the identification of yet more tandem repeat number variability to expand the Periscope Protein class. Within this class, structural and biophysical characterisation and molecular dynamics simulations of tandem repeat arrays will help to define the structure/function of these dynamic structural motifs. The functional implications of variability in repeat number are in general poorly understood, but the identification of strain-dependent repeat truncations and expansions in a broad array of proteins associated with bacterial niche adaptation and virulence highlights the need for more systematic studies into the mechanisms underpinning modulation of these dynamic bacterial surface proteins.

**Perspective**

- *Importance of the field*: Periscope Proteins underpin critical mechanisms in biofilm formation, host adhesion, immune evasion and virulence. Bacterial agility afforded by extension and retraction of this diverse protein class is currently sparsely researched, so drawing a spotlight on this functional grouping will help frame research questions to deepen our understanding of this mechanism of surface variability.
- *Summary of current thinking*: Identification and structure/function characterisation of the Periscope Protein class is revealing diverse protein folds, linkers and interdomain interfaces that form highly anisotropic linear arrays, supporting cell surface exposure of motifs for adhesion and multicellularity. This class contributes to microbial surface variation for bacterial niche adaptation with the potential for agile responses to selection pressures.
- *Future directions*: Recent bioinformatic identification of thousands of highly repetitive adhesive surface proteins highlights the need for research in this comparatively neglected bacterial landscape. Long-read sequencing initiatives are anticipated to reveal the breadth of the Periscope Protein class, framing key questions of spatial constraints at the bacterial cell surface, and how manipulation of repeat length contributes to niche adaptation and consortia establishment.

## Open Access

Open access for this article was enabled by the participation of University of Adelaide in an all-inclusive *Read & Publish* agreement with Portland Press and the Biochemical Society under a transformative agreement with CAUL.

## Abbreviations

Aap: accumulation associated protein
AsaA: associated with sialic acid adhesion A
E: extension of G5 domain
Fc: fragment crystallisable region
G5: G5 domain fold, five conserved glycine residues
GBS: group B *Streptococcus*
Ig: immunoglobulin
LytD: lytic enzyme D, *N*-acetylglucosaminidase
PATR: passenger-associated transport repeats
Pls: plasmin sensitive protein
Rib: resistance to proteases, immunity, group B
SasG: surface-associated protein G
Sgo0707: *Streptococcus gordonii* adhesin
SHIRT: Sgo0707 high identity repeats in tandem
Siglec: sialic-acid-binding immunoglobulin-like lectins
Spa: Staphylococcal protein A
SraP: serine-rich adhesin for platelets
TAA: trimerc autotransporter adhesin
